# Genome-wide prediction of vaccine targets for human herpes simplex viruses using Vaxign reverse vaccinology

**DOI:** 10.1186/1471-2105-14-S4-S2

**Published:** 2013-03-08

**Authors:** Zuoshuang Xiang, Yongqun He

**Affiliations:** 1 Unit for Laboratory Animal Medicine, University of Michigan Medical School, Ann Arbor, MI 48109, USA; 2 Department of Microbiology and Immunology, University of Michigan Medical School, Ann Arbor, MI 48109, USA; 3 Center for Computational Medicine and Bioinformatics, University of Michigan Medical School, Ann Arbor, MI 48109, USA; 4 Comprehensive Cancer Center, University of Michigan Medical School, Ann Arbor, MI 48109, USA

## Abstract

Herpes simplex virus (HSV) types 1 and 2 (HSV-1 and HSV-2) are the most common infectious agents of humans. No safe and effective HSV vaccines have been licensed. Reverse vaccinology is an emerging and revolutionary vaccine development strategy that starts with the prediction of vaccine targets by informatics analysis of genome sequences. Vaxign (http://www.violinet.org/vaxign) is the first web-based vaccine design program based on reverse vaccinology. In this study, we used Vaxign to analyze 52 herpesvirus genomes, including 3 HSV-1 genomes, one HSV-2 genome, 8 other human herpesvirus genomes, and 40 non-human herpesvirus genomes. The HSV-1 strain 17 genome that contains 77 proteins was used as the seed genome. These 77 proteins are conserved in two other HSV-1 strains (strain F and strain H129). Two envelope glycoproteins gJ and gG do not have orthologs in HSV-2 or 8 other human herpesviruses. Seven HSV-1 proteins (including gJ and gG) do not have orthologs in all 40 non-human herpesviruses. Nineteen proteins are conserved in all human herpesviruses, including capsid scaffold protein UL26.5 (NP_044628.1). As the only HSV-1 protein predicted to be an adhesin, UL26.5 is a promising vaccine target. The MHC Class I and II epitopes were predicted by the Vaxign Vaxitop prediction program and IEDB prediction programs recently installed and incorporated in Vaxign. Our comparative analysis found that the two programs identified largely the same top epitopes but also some positive results predicted from one program might not be positive from another program. Overall, our Vaxign computational prediction provides many promising candidates for rational HSV vaccine development. The method is generic and can also be used to predict other viral vaccine targets.

## Background

The Herpesviridae are a family of DNA viruses that cause diseases in humans and various animals. Herpesviruses are the members of the Herpesviridae family. All herpesviruses share a similar virion structure: a linear, double-stranded DNA molecule densely packaged into an icosahedral protein cage called capsid. The capsid is surrounded by an amorphous protein layer, called the tegument, consisting of both viral proteins and viral mRNAs and a lipid bilayer membrane (the envelope). Infectious virions are spherical. All herpesviruses are species-specific. Human herpesviruses (HHVs) include eight members: Herpes simplex virus (HSV) type 1 and 2 (HSV-1 and HSV-2), varicella zoster virus (VZV; HHV-3), Epstein-Barr virus (EBV; HHV-4), human cytomegalovirus (CMV; HHV-5), human herpesvirus-6 and -7 (HHV-6 and HHV-7), and Kaposi's sarcoma associated herpesvirus (KSHV; HHV-8). Herpesviruses typically cause latent, lytic, and recurring infections. HSV-1 and HSV-2 are two human pathogens that cause a variety of recurrent immunopathologic diseases, ranging from mild skin diseases including herpes labialis and herpes genitalis to life-threatening diseases including neonatal herpes and adult herpes encephalitis [1,2]. For example, HSV-1 can cause epithelial lesions on the lip or face. After establishment of productive infection, HSV-1 causes latent infection of the trigeminal ganglia. Despite fairly widespread use of antiviral drugs, HSV-1 and HSV-2 remain among the most common infectious agents of humans. In the US, the seroprevalence of HSV-1 and HSV-2 in adults is 68% and 21%, respectively; and approximately 700-2000 cases of neonatal HSV infections per year occur in the US [3].

Although many acute infections can be controlled by vaccination, the development of prophylactic and therapeutic vaccines against persistent herpesviruses remains challenging. There are currently no US FDA-approved HSV vaccines available. The development of an effective vaccine against HSV is complicated by many unique characteristics of herpes viruses, including the complexity of the virus replication cycle (i.e., primary, latent and recurrent phases of infection), their sophisticated immunoevasion strategies, a high number of protein candidates by the large and complex herpes genome [2]. Although antibodies generated following HSV-1 and HSV-2 immunizations do not protect against virus entry, antibodies against envelope glycoproteins gB, gC, gD, and gE provide passive protection against lethal viral challenges. T helper cell type 1 (Th1) response and cytotoxic T lymphocyte (CTL) activities are also critical to the host protection [4]. Many HSV proteins, including two major protective antigens gB and gD, have been evaluated for vaccine development [5,6]. Although animal studies showed induced protection, human clinical trials with vaccines using these two proteins (gB and gD) have not generated ideal results [5,6]. Therefore, for developing safe and effective human HSV vaccines, it is necessary to identify and evaluate more protective antigens in HSVs.

As an emerging and revolutionary vaccine development approach, reverse vaccinology starts with the prediction of vaccine protein targets by bioinformatics analysis of genome sequences [7]. Reverse vaccinology was first applied to development of a vaccine against serogroup B *Neisseria meningitidis *(MenB) [8]. With this method, it took less than 18 months to identify more protective vaccine targets in MenB than had been discovered during the past 40 years by conventional methods [8]. Afterwards, this technology has been successfully applied to many other pathogens such as *Bacillus anthracis *[9], *Streptococcus pneumoniae *[10], *Mycobacterium tuberculosis *[11], and *Cryptosporidium hominis *[12]. Vaxign is the first web-based vaccine design software that uses the reverse vaccinology strategy [13,14]. In reverse vaccinology, predicted proteins are selected based on defined desirable attributes. Predicted features in the Vaxign pipeline include protein subcellular location, transmembrane helices, adhesin probability, conservation among pathogenic strains, sequence exclusion from genomes of nonpathogenic strains, sequence similarity to host proteins, and epitope binding to Major histocompatibility complex (MHC) class I and class II. Vaxign has been demonstrated in successful prediction of verified and potential vaccine targets for *Brucella *spp. [14,15] and uropathogenic *E. coli *[13]. Over 200 genomes have been pre-computed using the Vaxign algorithm and available for query in the Vaxign website. Vaxign also allows for dynamic vaccine target prediction based on users' input sequences. Since the previous publication [13], Vaxign has included several new features. For example, after log in, a user can save Vaxign analysis projects for continuous updates and result sharing. While Vaxign includes its own MHC class I and II epitope prediction tool, Vaxign has now incorporated the tools implemented in the Immune Epitope Database (IEDB; http://tools.immuneepitope.org/main/html/tcell_tools.html). Both sets of epitope prediction results can then be compared in parallel.

The Vaxign reverse vaccinology approach is generic and can be used for analyses of vaccines against various pathogens and infection diseases. However, there has not been a report of how to use Vaxign to predict vaccine targets for a viral disease. In this study, we have used Vaxign to predict HSV vaccine targets.

## Results

### HSV genome data processing and Vaxign analysis

In total 52 herpesvirus genomes were identified from NCBI RefSeq and GenBank databases (Table 1). These genomes correspond to 52 herpesvirus strains from humans and different animal species. Twelve genomes come from human herpesviruses, covering all eight human herpesvirus types. This study aimed to predict herpes simplex virus vaccine targets, with an emphasis on HSV-1. HSV-1 has three strains including strain 17 (RefSeq ID: NC_001806.1), strain F (GenBank accession ID: GU734771.1), and strain H129 (GenBank accession ID: GU734772.1) (Table 1).

**Table 1:** Fifty-two herpesvirus genomes used in current Vaxign analysis.

#	Infected host	Herpesvirus strains	Genome #	No of proteins
**1**	Hartebeest	Alcelaphine herpesvirus 1	NC_002531.1	71
**2**	Anatids	Anatid herpesvirus 1	NC_013036.1	77
**3**	Anguillida	Anguillid herpesvirus 1	NC_013668.3	143
**4**	Atelinae	Ateline herpesvirus 3	NC_001987.1	73
**5**	Bovinae	Bovine herpesvirus 1	NC_001847.1	70
**6**	Bovinae	Bovine herpesvirus 4	NC_002665.1	79
**7**	Bovinae	Bovine herpesvirus 5	NC_005261.2	70
**8**	Callitrichidae	Callitrichine herpesvirus 3	NC_004367.1	72
**9**	Cavy	Caviid herpesvirus 2	NC_011587.1	106
**10**	Cercopithecinae	Cercopithecine herpesvirus 2	NC_006560.1	75
**11**	Cercopithecinae	Cercopithecine herpesvirus 9	NC_002686.2	74
**12**	Cyprinidae	Cyprinid herpesvirus 3	NC_009127.1	160
**13**	Horse	Equid herpesvirus 2	NC_001650.1	79
**14**	Horse	Equid herpesvirus 4	NC_001844.1	79
**15**	Horse	Equid herpesvirus 9	NC_011644.1	80
**16**	Cat	Felid herpesvirus 1	NC_013590.2	77
**17**	Chicken	Gallid herpesvirus 1	NC_006623.1	79
**18**	Chicken	Gallid herpesvirus 2	NC_002229.3	85
**19**	Chicken	Gallid herpesvirus 3	NC_002577.1	76
**20**	Human	**Human herpesvirus 1 strain 17**	NC_001806.1	77
**21**	Human	Human herpesvirus 1 strain F	GU734771.1	77
**22**	Human	Human herpesvirus 1 strain H129	GU734772.1	77
**23**	Human	Human herpesvirus 2 strain HG52	NC_001798.1	77
**24**	Human	Human herpesvirus 3	NC_001348.1	73
**25**	Human	Human herpesvirus 4	NC_009334.1	80
**26**	Human	Human herpesvirus 4 type 1	NC_007605.1	94
**27**	Human	Human herpesvirus 5	NC_006273.2	165
**28**	Human	Human herpesvirus 6A	NC_001664.2	88
**29**	Human	Human herpesvirus 6B	NC_000898.1	104
**30**	Human	Human herpesvirus 7	NC_001716.2	86
**31**	Human	Human herpesvirus 8	NC_009333.1	86
**32**	Catfish	Ictalurid herpesvirus 1	NC_001493.1	90
**33**	Macaque monkey	Macacine herpesvirus 1	NC_004812.1	75
**34**	Macaque monkey	Macacine herpesvirus 3	NC_006150.1	223
**35**	Macaque monkey	Macacine herpesvirus 4	NC_006146.1	80
**36**	Macaque monkey	Macacine herpesvirus 5, genome	NC_003401.1	89
**37**	Turkey	Meleagrid herpesvirus 1	NC_002641.1	79
**38**	Mouse	Murid herpesvirus 1	NC_004065.1	161
**39**	Mouse	Murid herpesvirus 2	NC_002512.2	167
**40**	Mouse	Murid herpesvirus 4	NC_001826.2	74
**41**	Oyster	Ostreid herpesvirus 1	AY509253.1	127
**42**	Sheep	Ovine herpesvirus 2	NC_007646.1	73
**43**	Panine	Panine herpesvirus 2	NC_003521.1	165
**44**	Mouse	Papiine herpesvirus 2	NC_007653.1	75
**45**	Psittacine birds	Psittacid herpesvirus 1	NC_005264.1	77
**46**	Ranidae	Ranid herpesvirus 1	NC_008211.1	132
**47**	Ranidae	Ranid herpesvirus 2	NC_008210.1	147
**48**	Rodent	Rodent herpesvirus Peru	NC_015049.1	82
**49**	Squirrel monkey	Saimiriine herpesvirus 1	NC_014567.1	70
**50**	Squirrel monkey	Saimiriine herpesvirus 2	NC_001350.1	76
**51**	Suidae	Suid herpesvirus 1	NC_006151.1	175
**52**	Tupaiid	Tupaiid herpesvirus 1	NC_002794.1	158

Vaxign is a vaccine design pipeline that assembles a list of separate software programs to calculate different criteria for individual proteins. For the prediction of viral vaccine targets, Vaxign allows a user to choose the following criteria:

(i) Sequence conservation among different genomes, e.g., among HSV-1 genomes or among all human herpesviruses.

(ii) Sequence exclusion from specific genomes, e.g., those HSV-1 proteins that do not have orthologous proteins in non-human herpesviruses.

(iii) Adhesin probability. Adhesin is critical for a virus to invade a host cell. So adhesins turn to be good vaccine targets.

(iv) The number of transmembrane helices. The presence of more than one transmembrane helix of a protein is often the result of failure of recombinant protein isolation and purification [13]. Therefore, a user can choose to not include those proteins with many transmembrane helices as possible vaccine targets.

(v) MHC Class I and II epitopes. A protein with many T cell epitopes is a preferred vaccine target. Also, prediction of MHC Class I and II epitopes is critical for those who plan to develop epitope vaccines.

Since different module software programs used in Vaxign are independent from each other, a user can choose whether or not to use any specific criteria and programs. Such module-based software pipeline is designed based on the observation that vaccine researchers and developers often have different preferences in terms which criteria to use and how to use them for their specific vaccine design applications. Below we present our predictions based on the schemes considered appropriate for development of HSV vaccines.

### Vaxign prediction of HSV protein vaccine targets

**Figure 1:**
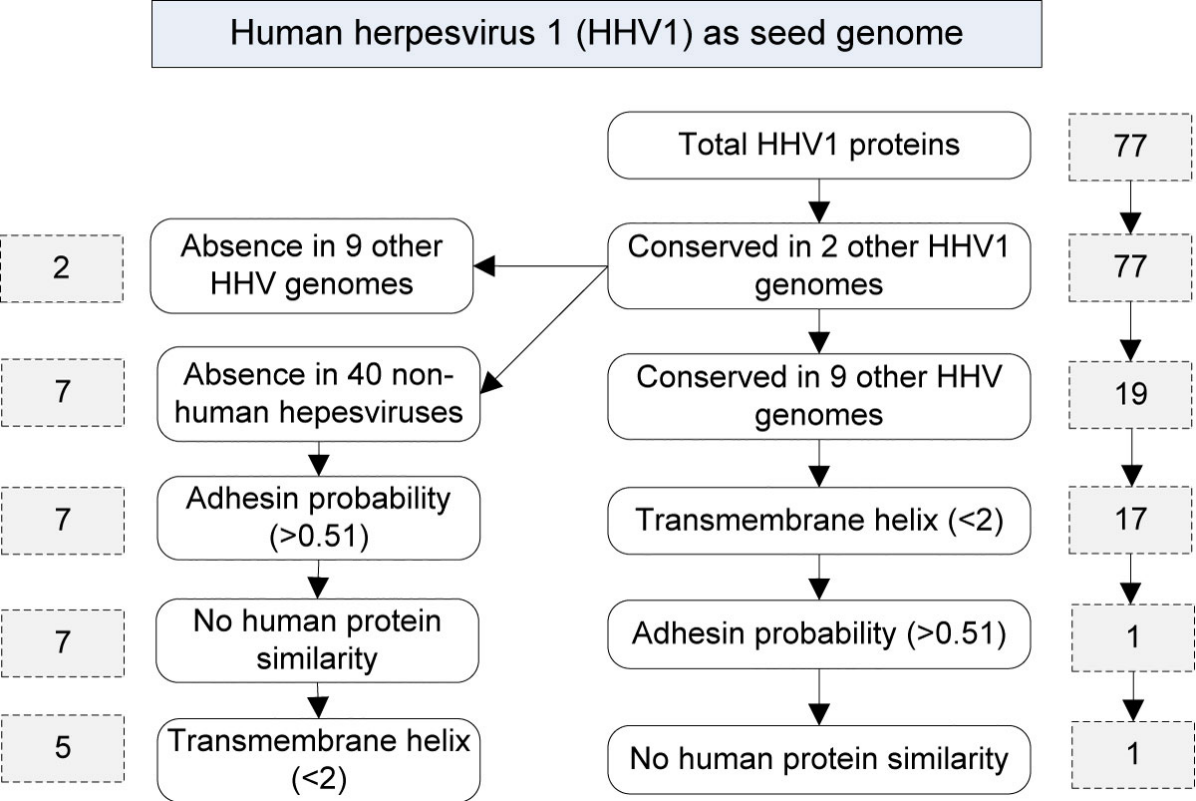
**The workflow and results of Vaxign prediction of HSV1 vaccine targets**. The highlighted human herpesvirus 1 strain 17 was used as the seed genome in the reported analysis.

The 52 herpesviruses into two groups: human herpesviruses and other animal herpesviruses. The human herpesviruses include three HSV-1 genomes, one HSV-2 genome, and eight human herpesvirus type 2-8 genomes. We have chosen to use HSV-1 (or HHV-1) strain 17 as the seed genome in our Vaxign analysis. The NCBI RefSeq genome annotation record shows that this genome includes 77 genes instead of the originally reported 72 genes [16]. The selection of this genome as a seed genome means that every chosen comparison among genomes is between the HSV-1 genome and other genome(s) and Vaxign reports the results using the HSV-1 proteins. HSV strain F and strain H129 were used for sequence conservation analysis. Our Vaxign analysis found that all 77 proteins in strain 17 also exist in HSV-1 strains F and H129 (Figure 1).

After the conservation analysis among all three HSV1 genomes, we have performed different Vaxign analyses using different schemes. First, our analysis identified two HSV-1 proteins that are conserved in all HSV-1 genomes but absent in all 9 genomes from seven other human herpesvirus types. These two proteins are envelope glycoprotein gJ (NP_044667.1) and envelope glycoprotein gG (NP_044666.1). The HSV-2 genome (NC_001798.1) is very similar to HSV-1 [17]. However, these two proteins gJ and gG do not have orthologs in HSV-2. Therefore, gJ and gG are likely critical to differentiate HSV-1 from HSV-2 and other human herpesviruses.

Seven out of 77 HSV-1 proteins were found absent in 40 non-human herpesviruses (Table 2). These seven proteins may differentiate HSV-1 from all other non-human herpesviruses. Since herpesviruses are very species-specific, the differentiation of orthologous proteins among human and non-human herpesviruses provides a way to identify the mechanism of host specificity of the viruses. These proteins may also be valuable to generate human-specific HSV-1 vaccines. However, an animal experiment with vaccine candidates using these proteins may not work out due to the host specificity issue. Among the seven proteins are two envelope glycoproteins gJ and gG, neurovirulent proteins NP_044661.1 and NP_044600.1 (Note: they are paralogs), transporter associated with antigen presentation (TAP) inhibitor ICP47, tegument protein US11, and membrane protein UL56. The two envelope glycoproteins were predicted to have two transmembrane helices each. UL56 was predicted to have one trans-membrane helix, which is consistent with the nature of UL56 being a membrane protein. None of these proteins have a favorable adhesin probability, indicating that they are not likely adhesins.

**Table 2:** Seven HSV-1 proteins that do not have orthologs in all 40 non-human herpesviruses.

#	Protein Accession	Protein Note	Adhesin Probability	Trans-membrane helices
**1**	NP_044675.1	TAP transporter inhibitor ICP47	0.079	0
**2**	NP_044674.1	tegument protein US11	0.245	0
**3**	NP_044667.1	envelope glycoprotein gJ	0.176	2
**4**	NP_044666.1	envelope glycoprotein gG	0.419	2
**5**	NP_044659.1	membrane protein UL56	0.238	1
**6**	NP_044661.1	neurovirulence protein ICP34.5	0.228	0
**7**	NP_044600.1	neurovirulence protein ICP34.5	0.228	0

Among 77 HSV-1 proteins, 19 are conserved also among nine other human herpesvirus (HHV) genomes (Table 3). Among the 19 HSV-1 proteins, four are capsid proteins, two are envelope glycoproteins, and five are DNA packaging proteins. The two envelope glycoproteins have three and eight transmembrane helices. The capsid scaffold protein UL26.5 (NP_044628.1; http://www.ncbi.nlm.nih.gov/protein/NP_044628.1) is the only one of the 19 proteins that has an adhesin probability of >0.51. This suggests that UL26.5 is an adhesin HSV-1 protein.

**Table 3:** 19 HSV-1 proteins that are also conserved in other human herpesviruses.

#	Protein Accession	Protein GI	Protein Note	Adhesin Probability	Trans-membrane helices
1	NP_044603.1	9629382	uracil-DNA glycosylase	0.262	0
2	NP_044606.1	9629385	helicase-primase helicase subunit	0.115	0
3	NP_044655.1	9629434	helicase-primase primase subunit	0.163	0
4	NP_044607.1	9629386	capsid portal protein	0.241	0
5	NP_044620.1	9629399	major capsid protein	0.113	0
6	NP_044627.1	9629406	capsid maturation protease (UL26)	0.386	0
7	NP_044628.1	9629407	capsid scaffold protein UL26.5	**0.675**	0
8	NP_044611.1	9629390	envelope glycoprotein gM	0.244	8
9	NP_044629.1	9629408	envelope glycoprotein gB	0.229	3
10	NP_044613.1	9629392	deoxyribonuclease	0.203	0
11	NP_044616.1	9629397	DNA packaging terminase subunit 1	0.165	0
12	NP_044630.1	9629409	DNA packaging terminase subunit 2	0.188	0
13	NP_044626.1	9629405	DNA packaging tegument protein UL25	0.210	0
14	NP_044634.1	9629413	DNA packaging protein UL32	0.185	0
15	NP_044635.1	9629414	DNA packaging protein UL33	0.264	0
16	NP_044625.1	9629404	nuclear protein UL24	0.195	0
17	NP_044631.1	9629410	single-stranded DNA-binding protein	0.168	0
18	NP_044632.1	9629411	DNA polymerase catalytic subunit	0.101	0
19	NP_044641.1	9629420	ribonucleotide reductase subunit 1	0.193	0

### Vaxign prediction of MHC Class I and II epitopes

Vaxitop is a Vaxign program that predicts MHC Class I and II binding epitopes based on position specific scoring matrices (PSSM) [13]. Currently many software programs for predicting T cell MHC Class I and II epitopes are available [18]. One unique feature about Vaxitop is that it reports a statistics P-value while other MHC Class I and II prediction tools typically report a percentage or top number. It has been recognized that an incorporation of different programs would increase the specificity of T cell epitope prediction. Therefore, we have installed the default IEDB MHC Class I and II prediction tools (http://tools.immuneepitope.org/main/html/tcell_tools.html) in Vaxign. A Vaxign user is allowed to calculate and compare the immune epitopes by using both Vaxitop and IEDB programs.

The addition of epitope prediction allows further analysis for the existence of potential HSV vaccine targets. Our analysis found that the HSV-1 UL26.5 capsid scaffold protein (NP_044628.1) [19] is particularly interesting. The adhesin probability of this protein is 0.675, which is the only protein that has an adhesin probability of more than 0.51, the default cutoff value of defining a predicted adhesin. We have thus focused here on the immune epitope predictions based on this capsid scaffold protein.

**Figure 2:**
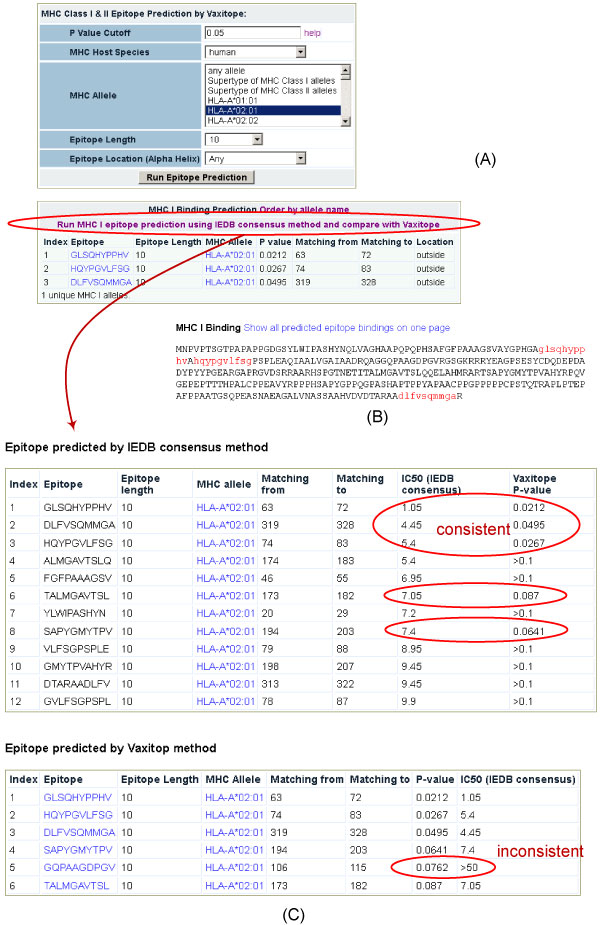
Vaxign prediction of MHC Class I HLA-A*0201 immune epitopes of HSV-1 UL26.5 capsid scaffold protein using Vaxign-Vaxitop program and IEDE prediction program. (A) Selection of parameters for epitope prediction. (B) Display of the locations of all predicted epitopes in the whole length protein. (3) Comparison of predicted results between Vaxitop and IEDB program. The label "consistent" means that the circled results were predicted by both programs. The label "inconsistent" indicates that the circled result was predicted to be positive by Vaxitop but not by IEDB program.

Our analyses found that the predicted results from the Vaxitop and IEDB MHC Class I prediction program usually overlap. Figure 2 provided an example when 10 amino acid epitopes were predicted for human HLA*A0201 allele. In this case, Vaxitop predicted three epitopes with a P-value cutoff value of 0.05 (Figure 2B and 2C) and six epitopes with a P-value cutoff of 0.1. IEDB predicted 12 epitopes with an IC50 cutoff of 10 and 109 epitopes for the same allele. Both programs predicted the same top three hits GLSQHYPPHV, HQYPGVLFSG, and DLFVSQMMGA. The Vaxitop P-value score for top 1 epitope GLSQHYPPHV is 0.0212. The IEDB IC50 value is 1.05. Among the six epitopes, five but one epitope was predicted by the IEDB program to have an IC50 value of < 50. The results show that these two programs predict similar top results but the top results may differ slightly. In general, Vaxign-Vaxitop is more conserved in producing the number of predicted epitopes than the IEDB program within the current cutoffs of these two programs. It is noted that we cannot provide evidence that the other top-score hits predicted by IEDB but not by Vaxitop are not significant.

Similarly, overlaping results could be found in MHC Class II epitope prediction between the results predicted from Vaxitop and the IEDB method (data not shown). The results of our receiver operating characteristic (ROC) curve data analyses using the positive and negative training data obtained from the IEDB database were comparable to the top results from existing MHC Class II prediction tools that were surveyed by Wang et al [28]. The results of the ROC analyses are provided in the Vaxign web page (http://www.violinet.org/vaxign/docs/aucs.php).

## Discussion

One major bottleneck for developing an effective and safe human HSV vaccine(s) is to identify protective antigens that are conserved among all HSV genomes and are able to induce protective immune response. Our current study is the first time to use a reverse vaccinology strategy to analyze various herpesvirus genomes and identify possible HSV vaccine targets based on genome sequence analyses. Our Vaxign reverse vaccinology approach has proven to be an efficient method to predict many valuable vaccine targets that are conserved in HSV genomes and contain desired characteristics.

Current study provides many vaccine targets for HSV vaccine development, including seven HSV-1 proteins that do not have orthologs in all tested non-human herpesviruses (Table 2). Among them are envelope glycoprotein gJ and gG. Antibody against gG has been found in HSV-1 infected individuals' serum samples [20]. HSV-1 gJ plays an important role in neuron-to-neuron transmission through synaptically linked neuronal pathways [21]. The membrane protein UL56 is likely involved in vesicular trafficking in HSV-infected cells [22]. HSV-1 ICP34.5 protein is a neurovirulence factor that plays critical roles in viral replication and anti-host responses [23]. HSV-1 ICP47, one of the seven proteins, is an early expressed protein that blocks the MHC class I antigen presentation pathway by binding to the TAP transporter [24]. These HSV proteins that do not have orthologs in non-human herpesviruses may be valuable human vaccine targets.

We have also found 19 HSV-1 proteins that are also conserved in other human herpesviruses (Table 3). This list includes two envelope glycoproteins gM and gB, five DNA packaging related proteins, and four capsid related proteins. Our analysis has identified capsid scaffold protein UL26.5 as a promising vaccine target. The primary reason is that UL26.5 is the only protein among all 77 HSV-1 proteins that has an adhesin probability of >0.51. The UL26.5 capsid scaffold protein is known to be critical for virus capsid formation [19]. During the assembly of a HSV capsid, the major capsid protein VP5 interacts with the C-terminal residues of the scaffold proteins UL26.5 and UL26 (also one of the 19 proteins in Table 3). After capsid assembly the scaffold proteins are cleaved at the maturation site by a serine protease also encoded by UL26, thereby allowing the scaffold proteins to be released from the capsid [25,26]. The cleaved UL26.5 protein releases the major scaffold protein VP22a. It is likely that the other cleaved segment plays a critical role in making UL26.5 a possible adhesin. An HSV-1 mutation with a deletion of UL26.5 amino acids 143-150 is unable to produce infectious virus [27]. It suggests that UL26.5 is a virulence factor critical for viral pathogenesis. UL26.5 is one of the 19 HSV-1 proteins that are also conserved in other human herpesviruses (Table 3). It means that this protein can be potentially used to develop a vaccine against all human herpesviruses. Experimental study is required to verify the value of UL26.5 or part of UL26.5 as a protective antigen for HSV vaccine development.

Various T cell MHC Class I and II epitope prediction algorithms have been developed and use different prediction approaches [18]. In general, T cell epitope algorithms have now achieved a high degree of prediction accuracy [28]. Different from other T cell epitope methods, Vaxitop uses a statistical P-value [13], which is more understandable to many biologists. Our comparative analysis found that Vaxitop predicted MHC Class I epitope results overlap with the IEDB prediction method and Vaxitop usually predicts less positive hits than the IEDB prediction (Figure 2). Since it is often that many epitopes are identified, it may be safe to experimentally test those epitopes that are positive from both predictions. The incorporation of these IEDB MHC Class I and II methods also provides the Vaxign users more options to perform immune epitope analysis.

The Vaxign vaccine design program can also be improved with additional features. For example, Vaxign lacks a program to predict the location of a viral protein inside the virion particle and its subcellular location inside host cells. Currently Vaxign includes PSORTb, a program that is designed for bacterial subcellular location prediction [29]. Another program for viral protein location would be needed, especially for those viruses containing a large genome. Since different criteria are provided in Vaxign, it is often a user's choice to balance the criteria for vaccine candidate selection. To make the selection more balanced, we are currently designing a comprehensive score that ranks predicted proteins by integrating different criteria. We are also in the process of incorporating gene expression profiles of microbial genes at different experimental conditions into our rational vaccine design. As an integrated component of the web-based VIOLIN database and analysis system (http://www.violinet.org) [30], Vaxign can also be improved by interaction with other VIOLIN programs. For example, VIOLIN Protegen is a web-based database that contains over 600 protective antigen information [31]. These experimentally verified protective antigens can be used for identifying specific patterns in protective antigens and computationally predicting protective antigens [32]. Many of the protective antigens in Protegen come from viruses, thus they can be used for the training of the Vaxign program and the verification of predicted results. The vaccine adjuvant database Vaxjo [33] in VIOLIN may provide training data for Vaxign to include a specific component for rational vaccine adjuvant design. The community-based Vaccine Ontology (VO; http://www.violinet.org/vaccineontology) is developed to support vaccine data standardization, integration, and computer-assisted reasoning [34]. VO has been found to be valuable in ontology-based natural language processing and literature mining [35], which can facilitate advanced vaccine design [36]. Currently, we are exploring how VO-based literature mining can improve Vaxign vaccine design.

## Methods

### Extraction and processing of herpesvirus genomes from NCBI

In total 52 herpesvirus genomes were retrieved from NCBI RefSeq [37] and GenBank [38] databases. The protein sequences were extracted and stored in the Vaxign relational database.

### Vaxign prediction of herpesvirus proteins as vaccine targets

For each herpesvirus protein, the Vaxign pipeline was used to calculate various criteria using module software programs described below:

**• Subcellular localization**. This feature is implemented in Vaxign using optimized PSORTb 3.0 [29]. PSORTb is the most precise bacterial protein subcellular localization predictor. To use this program, Vaxign first develops a script to generate standard input data for PSORTb. After the PSORTb execution, Vaxign automatically parses the PSORTb output and stores the results into the Vaxign MySQL database. Such a process allows seamless generation of PSORTb input, execution, and automatic processing and storage of PSORTb output in Vaxign. Similar strategies have been used in using other module programs.

**• Number of transmembrane helices**. The transmembrane helix topology analysis is conducted using optimized HMMTOP [14].

**• Minimum adhesin probability (0-1.0)**. Optimized SPAAN program [16] is used for calculating adhesin probability. A probability of greater than the default cutoff of 0.51 indicates that a tested protein is likely an adhesin or obtains adhesin-like characteristics. With this cutoff, the performance of the SPAAN program is optimized with the highest Matthews correlation coefficient[39].

**• Microbial sequence conservation by ortholog analysis**. OrthoMCL is applied for finding conserved proteins among a selected list of strains [17]. The E-value of 10^5 ^is set as the default value for OrthoMCL processing.

**• Exclusion of proteins having orthologs in selected genome(s)**. Similarly, OrthoMCL is applied for excluding proteins that also exist in a non-pathogenic strain(s).

**• No similarity to host proteins**. Choose this selection to exclude those vaccine targets that also exist in a host, including human, mouse, or pig.

### Vaxign-Vaxitop prediction of MHC Class I and II epitopes

Vaxign includes an internally developed program called Vaxitop, for prediction of MHC Class I & II epitopes. Vaxitop predicts immune epitopes based on position specific scoring matrices (PSSM). Different from other existing epitope prediction algorithms, Vaxitop calculates statistical P-value (instead of a percentage or top number) as the cutoff. A P-value of 0.05 provides a cutoff with high and balanced sensitivity and specificity [13]. We have also used Vaxitop to predict MHC Class I & II epitopes for each herpesvirus protein.

### Installation and incorporation of IEDB MHC Class I and II epitope prediction programs in Vaxign

As a new Vaxign feature, the IEDB MHC Class I and II epitope predictions programs have been downloaded from the IEDB website (http://tools.immuneepitope.org/main/html/tcell_tools.html). For each queried protein, these IEDB tools can be used to dynamically predict immune epitopes. The predicted results can be directly compared with the results output by Vaxitop.

### Query and analysis of herpesvirus vaccine targets using Vaxign web interface

After all proteins from 52 herpesvirus genomes were pre-computed, the results were made available for automatic query and deep analysis using the Vaxign web interface (http://www.violinet.org/vaxign).

## List of abbreviations

HHV: Human herpesvirus; HSV: Herpes simplex virus; IEDB: Immune Epitope Database; MHC: Major histocompatibility complex; MenB: Serogroup B *Neisseria meningitides*; NCBI: National Center for Biotechnology Information; PSSM: Position specific scoring matrices; TAP: Transporter associated with antigen presentation; US FDA: The United States Food and Drug Administration; VIOLIN: Vaccine Investigation and Online Information Network; VO: Vaccine Ontology.

## Competing interests

The authors declare that they have no competing interests.

## Authors' contributions

ZX: Data processing, Vaxign software programming, and database administrator.

YH: Project design, result analysis and interpretation, and manuscript writing.

## Declarations

The funding for publication of this article is provided by the NIH-NIAID grant R01AI081062 to YH.

This article has been published as part of *BMC Bioinformatics *Volume 14 Supplement 4, 2013: Special Issue on Computational Vaccinology. The full contents of the supplement are available online at http://www.biomedcentral.com/bmcbioinformatics/supplements/14/S4
